# Systemic IgG responses to *Leishmania braziliensis*–derived extracellular vesicles vary across clinical forms of American tegumentary leishmaniasis and associate with early therapeutic outcome

**DOI:** 10.3389/fimmu.2026.1874413

**Published:** 2026-09-29

**Authors:** Fabio C. Peixoto, José M. Guimarães, Carlos A. Oliveira Junior, Ingrid L. M. de Souza, Thiago M. Cardoso, Augusto M. Carvalho, Edgar M. Carvalho, Dalila L. Zanette, Lucas P. Carvalho

**Affiliations:** 1 Clinical Research Laboratory (LAPEC), Gonçalo Moniz Institute (IGM), Oswaldo Cruz Foundation (FIOCRUZ), Salvador, Bahia, Brazil; 2 Post Graduation Program of Health Sciences (PPGCS), Federal University of Bahia (UFBA), Salvador, Bahia, Brazil; 3 Laboratory for Applied Sciences and Technology in Health, Carlos Chagas Institute, FIOCRUZ, Curitiba, Paraná, Brazil; 4 National Institute of Sciences and Technology – Tropical Diseases (INCT-DT), Salvador, Bahia, Brazil

**Keywords:** American tegumentary leishmaniasis, extracellular vesicles, humoral immune response, IgG, *L. braziliensis*

## Abstract

**Background:**

American tegumentary leishmaniasis (ATL) caused by Leishmania braziliensis is characterized by marked clinical and immunopathological heterogeneity. While cellular immune responses have been extensively investigated, the contribution of humoral immunity, particularly against parasite-derived extracellular vesicles (EVs) components, remains poorly understood. Here, we evaluated systemic IgG responses against EVs derived from L. braziliensis isolates associated with distinct clinical forms of ATL and assessed their association with disease presentation and therapeutic outcome.

**Methods:**

Serum samples from patients with cutaneous leishmaniasis (CL), mucosal leishmaniasis (ML), disseminated leishmaniasis (DL), early cutaneous leishmaniasis (ECL), and healthy subjects were analyzed by ELISA using intact EVs.

**Results and discussion:**

CL patients exhibited higher anti-EV IgG levels than healthy subjects, confirming the induction of EV reactive humoral responses during active disease. Comparative analysis revealed that CL and DL patients displayed higher anti-EV IgG levels than ML patients, whereas ECL patients exhibited lower levels than classical CL, consistent with reduced systemic humoral responses at early stages of infection. IgG responses against EVs derived from different isolates were strongly correlated, indicating shared antigenic determinants exposed on the vesicle surface. Higher baseline IgG levels against EV surface antigens were associated with clinical cure at day 60 following antimonial therapy but did not predict outcome at day 90, suggesting a relationship with early healing dynamics rather than definitive lesion resolution. Anti-DL EV IgG levels showed a weak correlation with time to lesion healing. In contrast, short-term in vitro stimulation of peripheral blood mononuclear cells with EVs did not alter circulating B cell subset distribution. Together, these findings demonstrate that L. braziliensis–derived EVs are immunologically relevant targets of systemic humoral responses in humans and that IgG reactive to EVs profiles reflect clinical form and early therapeutic response. EV-based serological analyses may therefore provide complementary insights into immune dynamics and disease progression in ATL.

## 1 Introduction

American tegumentary leishmaniasis (ATL), caused by protozoan parasites of the genus *Leishmania*, remains a major public health problem in endemic regions worldwide, including Brazil. Infection is transmitted by the bite of infected sand flies, and parasites primarily infect macrophages and dendritic cells, giving rise to a wide spectrum of clinical manifestations. These include localized cutaneous leishmaniasis (CL), disseminated leishmaniasis (DL), mucosal leishmaniasis (ML), and diffuse cutaneous leishmaniasis (DCL), each associated with distinct immunopathological profiles (1–3). In Brazil, CL is predominantly caused by *Leishmania (Viannia) braziliensis* and is characterized by one or a few ulcerated skin lesions with raised borders and granulomatous bases (3).

Despite similar clinical appearances, ATL caused by different *Leishmania* species — and even by distinct strains of *L. braziliensis* — exhibits marked differences in parasite burden, immune responses, and clinical outcome. In contrast to infections caused by species such as *L. mexicana*, *L. guyanensis*, or *L. major*, where parasites are abundant in lesions, parasites are scarce or often undetectable in *L. braziliensis*–associated disease (4–7). Importantly, *L. braziliensis* infection may progress beyond localized cutaneous disease, leading to severe forms such as ML and DL, which are associated with increased morbidity and therapeutic challenges (8, 9).

Host immune responses play a central role in disease outcome. In CL caused by *L. braziliensis*, parasite control is associated with a robust Th1-type immune response characterized by high production of IFN-γ and TNF and low levels of IL-10 (10–12). While this inflammatory response contributes to parasite restriction, it is insufficient to achieve sterile cure. Persistent antigenic stimulation leads to sustained immune activation, excessive inflammation, and tissue damage, ultimately contributing to ulcer formation and delayed healing (13–17). Indeed, patients with ML and atypical or disseminated forms of disease display heightened lymphocyte proliferation and increased production of pro-inflammatory mediators compared to individuals with classical CL, highlighting the close association between immune activation and disease severity (10, 11, 18, 19).

Although the role of innate and T cell–mediated immunity in ATL has been extensively studied, the contribution of humoral immune responses to disease pathogenesis and clinical outcome remains incompletely understood. Elevated levels of anti-*Leishmania* antibodies are detected across all clinical forms of leishmaniasis, with particularly high IgG and IgE titers observed in patients with ML and severe disease (20). Moreover, increased anti–soluble *Leishmania* antigen (SLA) IgG levels have been associated with disease severity and poor clinical outcome, including therapeutic failure (20, 21). These observations suggest that humoral responses may reflect ongoing antigenic stimulation and immune dysregulation rather than protective immunity.

Extracellular vesicles (EVs), including exosomes, have emerged as key mediators of host–pathogen interactions. These nano-sized vesicles (30–200 nm) are released by both host cells and parasites and transport bioactive cargo such as proteins, lipids, and nucleic acids capable of modulating recipient cell function (22–26). In *Leishmania*, EVs have been shown to deliver virulence factors, including GP63, to host macrophages, altering signaling pathways and exacerbating inflammatory responses in experimental models (22, 25–28). Importantly, EV cargo composition varies according to parasite species, strain, and developmental stage, suggesting a potential role in shaping distinct immune responses and clinical outcomes (29–32).

Previous studies from our group have demonstrated that *L. braziliensis*–derived EVs induce robust pro-inflammatory responses in human immune cells, including enhanced production of IL-1β, IL-6, IFN-γ, and TNF (33). However, whether EVs also elicit specific humoral immune responses in infected individuals — and whether such responses are associated with disease severity or therapeutic outcome — remains largely unexplored.

Given the persistent antigenic stimulation characteristic of *L. braziliensis* infection and the strong immunogenic potential of parasite-derived EVs, we hypothesized that IgG responses against EVs may reflect distinct clinical forms of ATL and could serve as biomarkers of disease severity and treatment response. In the present study, we investigated systemic IgG responses against EVs derived from *L. braziliensis* isolates associated with CL, ML, and DL. We further evaluated the association between anti-EV IgG levels, clinical presentation, and therapeutic outcome in patients with ATL. Our findings reveal differential humoral responses among EVs across clinical forms of disease and support a role for parasite-derived EVs as immunologically relevant targets in human leishmaniasis.

## 2 Materials and methods

### 2.1 Study design

This was an observational study designed to investigate the humoral immune response against *Leishmania braziliensis*–derived EVs and its association with clinical presentation and therapeutic outcome in patients with ATL. Serum levels of IgG antibodies against EVs derived from *L. braziliensis* isolates associated with different clinical forms were evaluated in patients with CL, ML, DL, ECL. In a subgroup of CL patients, the predictive value of anti-EV IgG levels for therapeutic response was further assessed in a longitudinal analysis.

### 2.2 Study subjects

Patients were recruited from the endemic area of Corte de Pedra, Bahia, Brazil, an area with intense transmission of *Leishmania braziliensis*. A total of patients with CL (n=72), ML (n=11), DL (n=41), and ECL (n=19) were enrolled, as well as healthy subjects (HS), residents of a leishmaniasis non-endemic area. Diagnosis of ATL was established based on compatible clinical presentation and confirmation of *L. braziliensis* infection by polymerase chain reaction (PCR) performed on lesion samples.

CL was defined by the presence of one or a few well-delimited ulcerated skin lesions. ML was defined by involvement of nasal, oral, or pharyngeal mucosa, with or without a previous history of cutaneous disease. DL was characterized by the presence of ten or more acneiform, papular, or ulcerated lesions distributed over two or more noncontiguous body regions. ECL was defined by non-ulcerated papular or nodular lesions with disease duration shorter than 30 days.

Inclusion criteria were age between 18 and 60 years and confirmed *L. braziliensis* infection. Exclusion criteria included pregnancy, diabetes mellitus, obesity, autoimmune or inflammatory diseases, immunosuppressive conditions, and chronic renal or hepatic disease.

All CL patients included in the longitudinal analysis were treated with pentavalent antimony (meglumine antimoniate, Glucantime^®^, Sanofi-Aventis) at a dose of 20 mg/kg/day administered intravenously for 20 consecutive days, with a maximum daily dose of 1,200 mg. Therapeutic outcome was defined as clinical cure, characterized by complete re-epithelialization of skin lesions, or therapeutic failure, defined by incomplete healing, assessed at 60 (D60) and 90 (D90) days after initiation of treatment.

Serum samples were collected prior to the initiation of therapy (day 0) from all patient groups. Serum samples from healthy subjects (HS) living in non-endemic areas were used as controls for antibody analyses.

### 2.3 Parasite culture

*Leishmania braziliensis* isolates obtained from patients with CL (MHOM/BR/LTCP30833), ML (MHOM/BR/LTCP26060), and DL (MHOM/BR/LTCP30895) were initially cultured in biphasic Novy–MacNeal–Nicolle (NNN) medium. Parasites were identified by multilocus enzyme electrophoresis and subsequently cryopreserved in liquid nitrogen. For experimental procedures, parasites were expanded in Schneider’s insect medium (Sigma-Aldrich) supplemented with 20% heat-inactivated fetal bovine serum (FBS), 1% L-glutamine, and antibiotics.

### 2.4 Isolation of *L. braziliensis* extracellular vesicles

Promastigotes from each isolate were cultured at 37 °C and 5% CO_2_ for 4 hours in FBS-free Schneider’s medium to optimize EV release. Culture supernatants were sequentially centrifuged to remove parasites and debris, followed by filtration through 0.22-µm membranes. EVs were isolated by ultracentrifugation at 100,000 × g and washed in sterile phosphate-buffered saline (PBS).

### 2.5 Characterization of *L braziliensis* extracellular vesicles

EVs were characterized and quantified as previously described (34). Briefly, EVs samples were diluted 1:4 in PBS to a final syringe volume of 1 mL and analyzed with a Nanosight LM14C instrument (Malvern Instruments, Malvern, UK). The ideal measurement concentration was 20–100 particles/frame. The following settings were used: the camera level was increased until all particles were distinctly visible, not exceeding a particle signal saturation of 20% (cell line-derived EVs: camera levels between 10-12). The ideal detection threshold was determined to include as many particles as possible within a range of 50–200 red crosses. Additionally, blue crosses were limited to 5. Autofocus was adjusted so that indistinct particles were avoided. For each measurement, three videos of 60s were captured under the following conditions: 25 °C cell temperature; infusion rate at 1000 units; and syringe pump speed at 100 µl/s. After capture, the videos were analyzed by the NanoSight Analytical Software—NTA 3.4, Build 3.4.4, with a detection threshold of 2 or 3, in order to exclude unspecific non-EVs particles (**Figure 1**).

**Figure 1:**
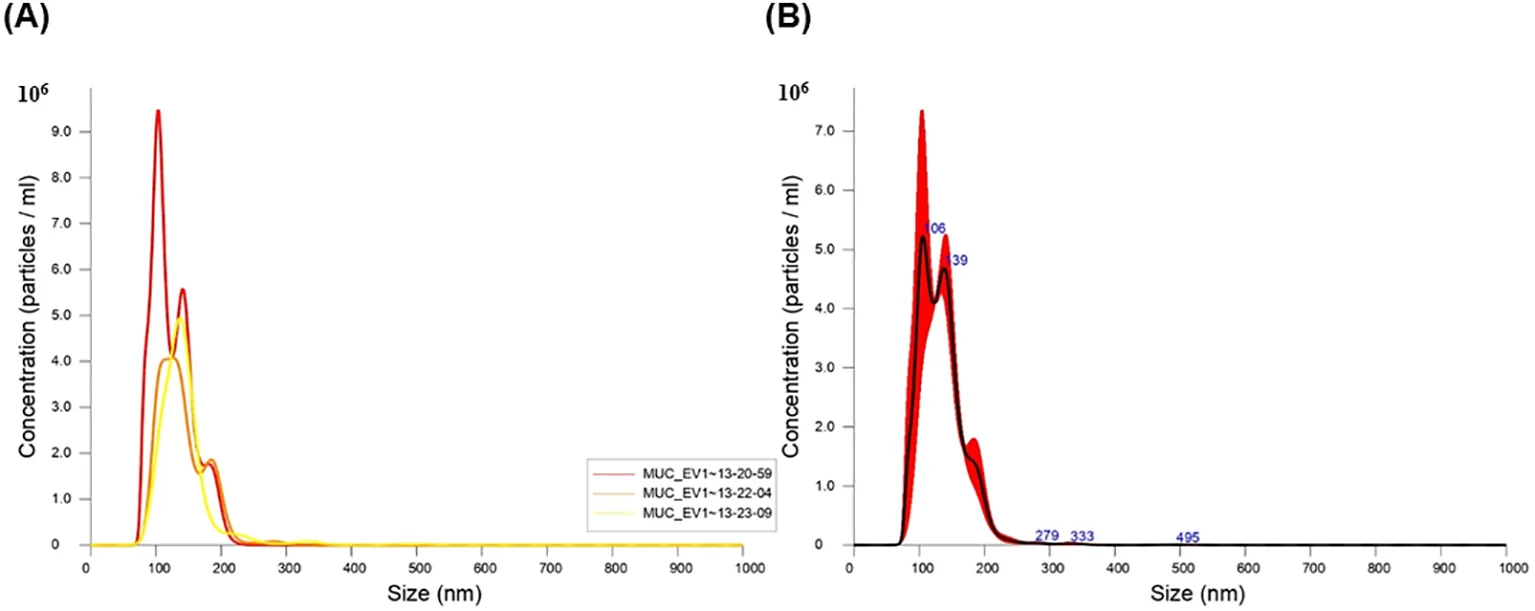
Characterization of *Leishmania braziliensis* extracellular vesicles (EVs) by nanoparticle tracking analysis (NTA). **(A)** Size distribution profiles from three technical recordings of isolated EVs derived from isolates associated with cutaneous, mucosal, and disseminated forms of American tegumentary leishmaniasis. **(B)** FTLA-corrected average size distribution demonstrating enrichment of vesicles predominantly within the small EV size range (~100–200 nm). The mean particle concentration was 3.85 × 10^8^ particles/mL.

Total protein content was quantified by the bicinchoninic acid (BCA) assay, following the manufacturer’s instructions. Absorbance was measured using a microplate reader, and protein concentrations were calculated based on a standard curve generated with bovine serum albumin (BSA).

### 2.6 Soluble *Leishmania* antigen

Soluble *Leishmania* antigen (SLA) was prepared from an *L. braziliensis* isolate obtained from a patient with ML, as previously described. Parasites were disrupted by sonication, and insoluble material was removed by centrifugation. Protein concentration was determined by the Bradford assay, and endotoxin levels were assessed using the Limulus amebocyte lysate test.

### 2.7 Detection of anti-EV and anti-SLA IgG antibodies

Serum IgG antibodies against EVs derived from CL, ML, and DL isolates, as well as against SLA, were quantified by enzyme-linked immunosorbent assay (ELISA). Briefly, polystyrene plates were coated overnight at 4 °C with 20 µg/well of EVs or SLA diluted in carbonate–bicarbonate buffer. Plates were blocked with PBS containing 10% bovine serum albumin and incubated with patient or control sera diluted 1:50. Bound antibodies were detected using horseradish peroxidase–conjugated anti-human IgG, followed by development with tetramethylbenzidine substrate. Optical density was measured at 450 nm.

Cutoff values for seropositivity were determined by receiver operating characteristic (ROC) curve analysis based on absorbance values obtained from healthy subjects.

### 2.8 Peripheral blood mononuclear cell isolation and stimulation

Peripheral blood mononuclear cells (PBMCs) were isolated from heparinized blood by density-gradient centrifugation using Ficoll-Paque. Cells were washed, resuspended in RPMI 1640 supplemented with 10% FBS and antibiotics, and adjusted to a final concentration of 3 × 10^6^ cells/mL.

PBMCs from CL patients were stimulated with EVs derived from CL, ML, or DL isolates (10µg/mL) in the presence or absence of SLA (5 µg/mL) and cultured for 72 hours at 37 °C and 5% CO_2_. Cells were harvested for flow cytometric analysis.

### 2.9 B cell phenotyping

PBMCs were stimulated with EVs from different *L. braziliensis* isolates in the presence or absence of SLA. After incubation, cells were treated with GolgiStop™, stained with fluorochrome-conjugated antibodies against CD19, CD24, CD38, and IL-10, and analyzed by flow cytometry. Data acquisition was performed using a BD LSRFortessa™ cytometer, and analyses were conducted using FlowJo™ software.

### 2.10 SDS-PAGE and silver staining

Protein extracts were quantified using the Pierce™ BCA Protein Assay Kit (Thermo Fisher Scientific, Waltham, MA, USA), and equal amounts of total protein (100 μg per lane) were loaded onto the gels. Samples were mixed with Laemmli sample buffer containing sodium dodecyl sulfate (SDS) and heat-denatured at 90 °C for 5 min before electrophoresis.

Protein separation was performed by sodium dodecyl sulfate–polyacrylamide gel electrophoresis (SDS-PAGE) using the SureCast™ Handcast Gel System (Thermo Fisher Scientific, Waltham, MA, USA). Gels consisted of a 10% resolving gel and a 4% stacking gel, prepared according to the manufacturer’s instructions. A Thermo Scientific™ Spectra™ Multicolor Broad Range Protein Ladder was used as the molecular weight standard. Electrophoresis was carried out in Tris-Glycine-SDS running buffer under constant voltage conditions until adequate protein separation was achieved.

Following electrophoresis, proteins were visualized using the PlusOne™ Silver Staining Kit (GE Healthcare, Uppsala, Sweden) according to the manufacturer’s protocol. Briefly, gels were fixed, sensitized, silver impregnated, developed, and treated with stop solution. After staining, gels were rinsed with ultrapure water and digitally scanned at high resolution. Protein banding patterns and molecular weight distributions were subsequently analyzed and compared among the different protein extracts and fractions obtained from *Leishmania (Viannia) braziliensis* clinical isolates.

### 2.11 Statistical analysis

Statistical analyses were performed using GraphPad Prism software (version 8.0.2). Comparisons between two independent groups were conducted using the Mann–Whitney U test, while paired data were analyzed using the Wilcoxon signed-rank test. Multiple group comparisons were performed using the Kruskal–Walli’s test followed by Dunn’s post-test. Correlations were evaluated using Pearson’s rank correlation coefficient. ROC curve analysis was used to assess the diagnostic and predictive performance of anti-EV IgG levels. A p value < 0.05 was considered statistically significant.

## 3 Results

### 3.1 CL patients exhibit elevated systemic IgG responses against *L. braziliensis* EVs

To initially determine whether *L. braziliensis* EVs elicit systemic humoral immune responses during active disease, serum IgG levels against EVs were quantified in patients with cutaneous leishmaniasis (CL) and compared to healthy subjects (HS) from non-endemic areas.

Patients with CL displayed significantly higher serum levels of IgG antibodies against EVs derived from CL, ML, and DL *L. braziliensis* isolates when compared to HS (**Figure 2a**). Similar results were observed for anti–soluble *Leishmania* antigen (SLA) IgG, which was used as a positive control. These findings demonstrate that infection with *L. braziliensis* induces systemic IgG responses against parasite-derived EVs in patients with active cutaneous disease.

**Figure 2:**
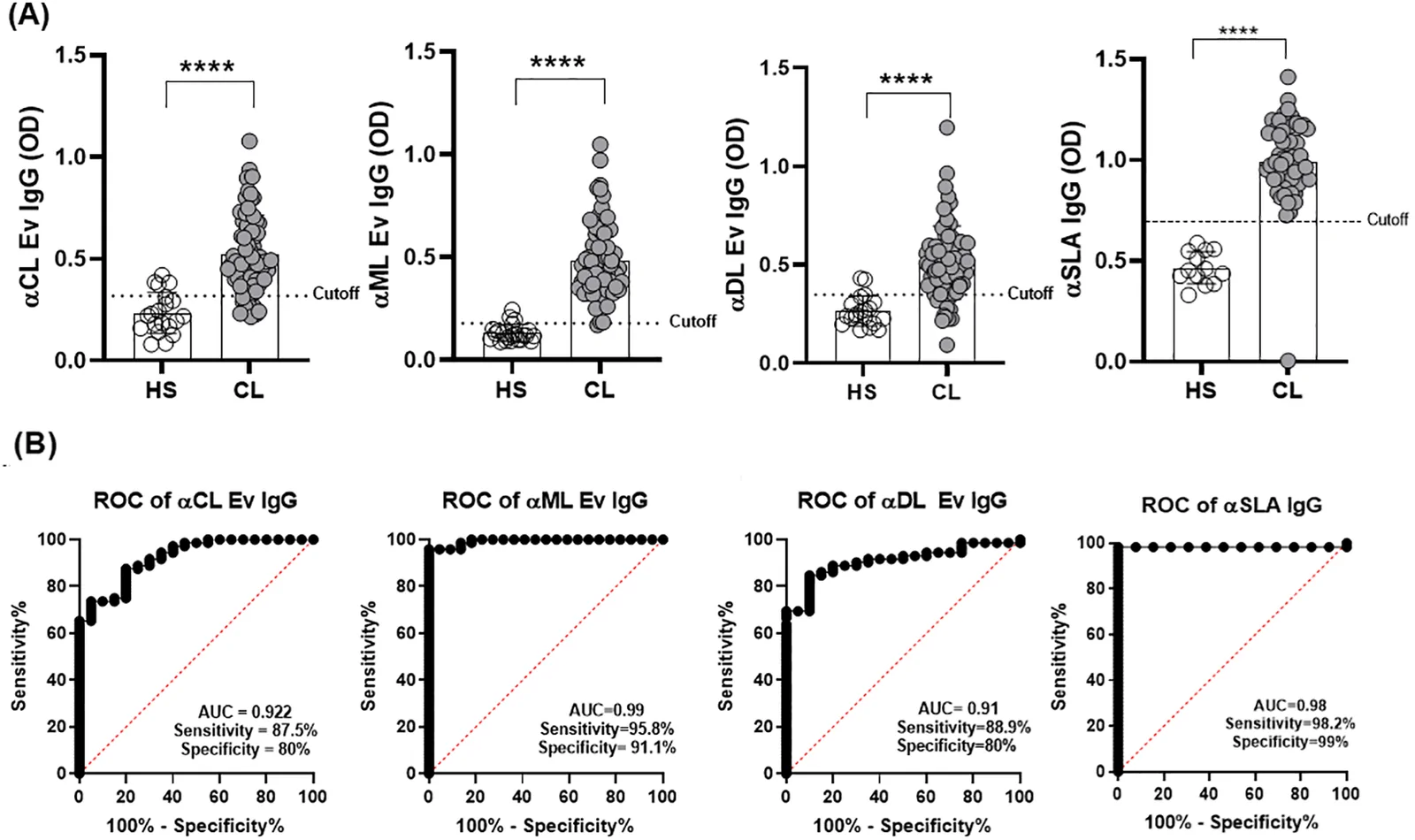
Serum anti–extracellular vesicle IgG levels discriminate cutaneous leishmaniasis patients from healthy subjects. **(A)** Serum IgG levels directed against EVs derived from *Leishmania braziliensis* isolates associated with CL, ML and DL were quantified in patients with CL (n = 72) and compared to healthy subjects (HS; n = 20). IgG responses against soluble *Leishmania* antigen (SLA) were included as a positive control. Data are shown as individual values with median and interquartile range. **(B)** Receiver operating characteristic (ROC) curve analyses corresponding to αCL EV IgG, αML EV IgG, αDL EV IgG and αSLA IgG are presented in the lower panels. Statistical analyses were performed using nonparametric Mann-Whitney U test (two groups) and Kruskal-Wallis + Dunn’s post-test (multiple groups). *p* < 0.05, **** p<0.0001.

Receiver operating characteristic (ROC) curve analysis revealed that anti-EV IgG levels efficiently discriminated CL patients from HS (**Figure 2b**), displaying high sensitivity and specificity. However, when compared directly, SLA-based serology exhibited superior diagnostic performance, indicating that although EVs are strongly immunogenic, SLA remains a highly sensitive antigen for the serological detection of CL (**Figure 2b**).

### 3.2 Systemic anti-EV IgG responses differ among clinical forms of ATL

In a second and independent analysis, serum IgG responses against *L. braziliensis* EVs were compared across distinct clinical forms of ATL, including CL, ML, DL, and ECL.

Patients with CL and DL exhibited significantly higher levels of total IgG antibodies directed against EVs derived from CL, ML, and DL isolates when compared to patients with ML (**Figure 3**). Individuals with ECL also presented elevated anti-EV IgG levels, comparable to those observed in CL patients (**Figure 3**). In contrast, ML patients showed consistently lower systemic IgG reactivity against EVs, despite active disease.

**Figure 3:**
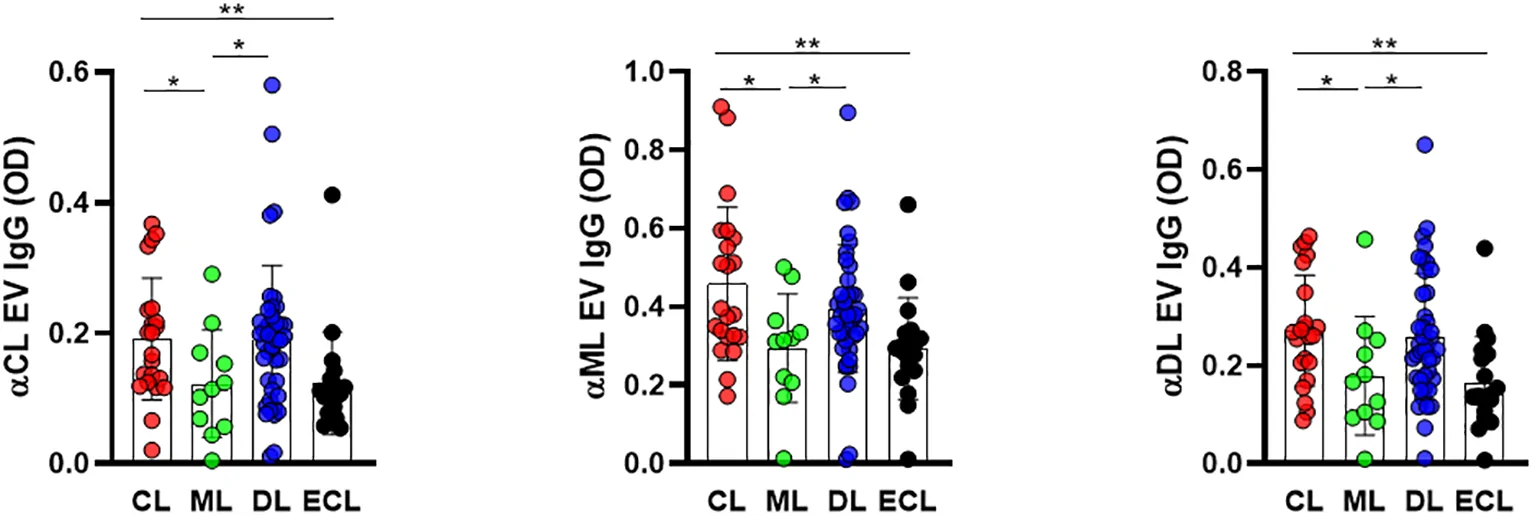
Differential serum anti–extracellular vesicle IgG responses across clinical forms of American tegumentary leishmaniasis. Serum IgG levels directed against extracellular vesicles (EVs) derived from *Leishmania braziliensis* isolates associated with cutaneous leishmaniasis (CL), mucosal leishmaniasis (ML), and disseminated leishmaniasis (DL) were quantified in patients with CL (n = 22), ML (n = 11), DL (n = 41), and early cutaneous leishmaniasis (ECL; n = 19). Data are shown as individual values with median and interquartile range. Statistical analyses were performed using nonparametric Mann-Whitney U test (two groups) and Kruskal-Wallis + Dunn’s post-test (multiple groups). *p* < 0.05, *p<0.05, **p<0.01.

These results indicate that humoral immune responses against EVs vary according to the clinical form of ATL and suggest that systemic exposure to EV antigens is more pronounced in cutaneous and disseminated disease than in mucosal involvement.

### 3.3 IgG anti-EVs responses from CL patients correlate across vesicles derived from isolates associated with distinct clinical forms of ATL

To evaluate whether humoral responses against EVs derived from different *L. braziliensis* isolates reflected shared antigenic determinants, correlations between serum IgG levels against CL EVs, ML EVs, and DL EVs were analyzed in CL patients.

A strong positive correlation was observed between IgG levels directed against CL EVs and ML EVs, CL EVs and DL EVs, and ML EVs and DL EVs (**Figure 4**). These findings are consistent with the hypothesis that EVs from different clinical form isolates share conserved antigenic components capable of eliciting systemic humoral responses.

**Figure 4:**
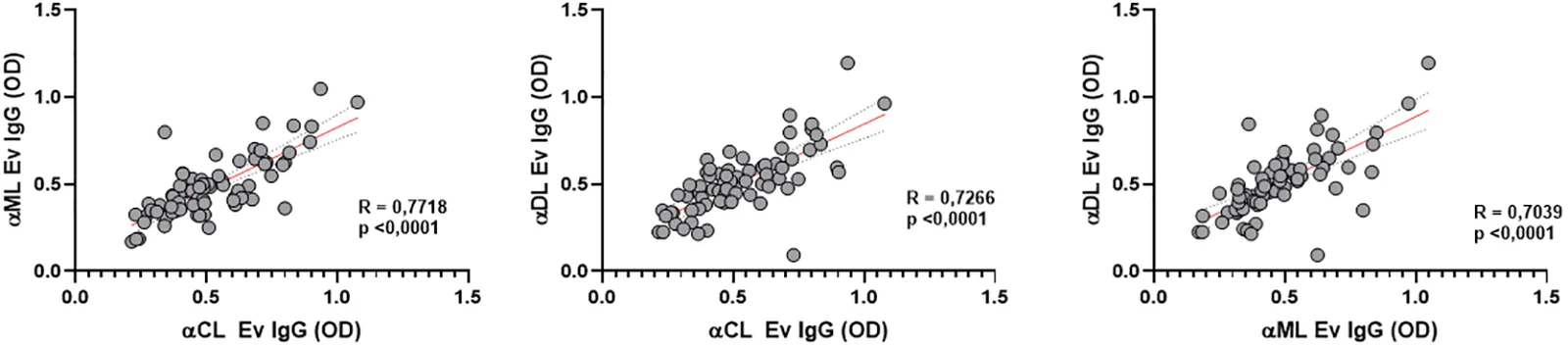
Correlation between CL serum IgG against EVs from different clinical forms of ATL. Pearson correlation of αCL EV IgG vs αML EV IgG, αCL EV IgG vs αDL EV IgG and αML EV IgG vs αDL EV IgG in the serum of CL patients (n=72).

### 3.4 Elevated anti-EV IgG levels are associated with poor early therapeutic outcome in CL patients

To determine whether humoral immune responses against *L. braziliensis* EVs were associated with therapeutic outcome, serum anti-EV IgG levels were analyzed in patients with CL prior to the initiation of antimonial therapy and stratified according to treatment response.

At day 60 after treatment initiation, patients who achieved clinical cure exhibited significantly lower levels of IgG antibodies against EVs derived from CL and ML isolates compared to patients who experienced therapeutic failure (**Figure 5a**). In contrast, no significant differences were observed in IgG levels directed against DL-derived EVs between cured and non-cured individuals. Receiver operating characteristic (ROC) curve analysis demonstrated moderate sensitivity and specificity for anti-CL EV and anti-ML EV IgG levels in predicting therapeutic failure at day 60 (**Figure 5b**).

**Figure 5:**
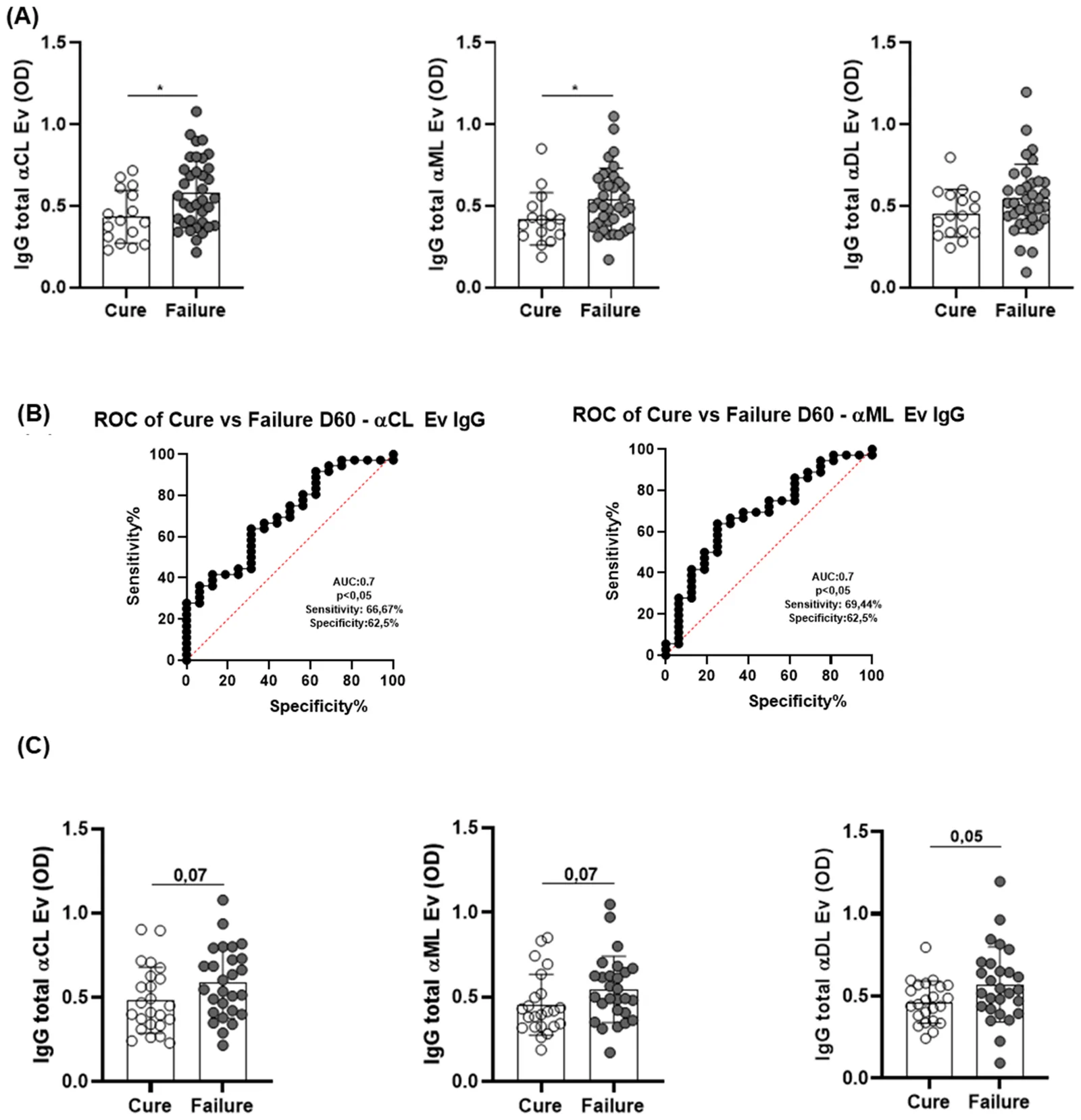
Serum levels of IgG αCL EV, αML EV and αDL EV from CL patients divided between those who cured and failed antimony therapy. **(A)** Serum levels of αCL EV IgG, αML EV IgG and αDL EV IgG from CL patients divided between those who cured (n=18) and failed antimony therapy (n=41), after 60 days of treatment. **(B)** ROC from levels of αCL EV IgG and αML EV IgG. **(C)** Serum levels of αCL EV IgG, αML EV IgG and αDL EV IgG from CL patients divided between those who cured and failed antimony therapy. Cure (n=25) and failure (n=33); treatment outcome was determined by the complete closure of the lesion on day 90 or not. Statistical analyzes were performed using nonparametric Mann-Whitney U test (two groups) and Kruskal-Wallis + Dunn’s post-test (multiple groups). * p <0.05.

When therapeutic outcome was evaluated at day 90 after treatment initiation, no significant differences in anti-EV IgG levels were observed between cured and non-cured patients for any EV preparation (**Figure 5c**). These findings indicate that EV driven humoral responses are associated with early therapeutic outcome but do not reliably predict long-term lesion healing.

### 3.5 Anti-DL EV IgG levels weakly correlate with time to lesion healing

To further investigate the clinical relevance of EV reactive humoral responses, correlations between serum anti-EV IgG levels and clinical parameters were evaluated in CL patients.

No significant correlations were observed between IgG levels directed against CL or ML EVs and lesion size, duration of illness, or time to healing (**Supplementary Data**). However, IgG levels against DL-derived EVs showed a weak but statistically significant positive correlation with time to lesion healing (**Supplementary Data**), indicating that elevated humoral responses against EVs associated with disseminated disease may reflect sustained antigenic exposure and prolonged inflammatory activity.

### 3.6 Comparative protein profiling of *L. braziliensis* EVs and SLA

To evaluate and compare the protein composition of our samples, we performed an SDS-PAGE analysis (**Figure 6**). The electrophoretic separation revealed a highly conserved protein profile among the CL, DL, and ML samples. A prominent hallmark across these three preparations is the presence of an intensely stained, well-defined double protein band migrating just above the 40 kDa molecular weight marker. This pronounced visual similarity indicates that the CL, DL, and ML fractions share a highly homologous protein composition, particularly regarding their most abundant structural components. In stark contrast, the Soluble *Leishmania* Antigen (SLA) exhibited a fundamentally distinct protein signature. The major double band characteristic of the CL, DL, and ML fractions is notably absent in the SLA lanes, which instead display a diffuse and faint pattern of sparsely distributed bands. The distinct electrophoretic pattern of SLA compared to other purified antigens is consistent with previously reported data in the literature, which corroborates this characteristic separation profile for SLA (35).

**Figure 6:**
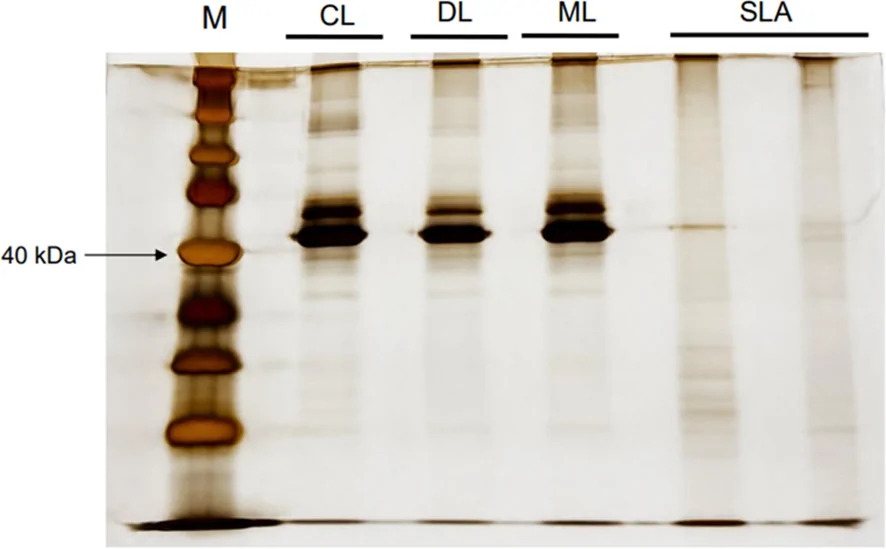
Comparative SDS-PAGE analysis of extracellular vesicle proteins derived from Leishmania braziliensis isolates associated with different clinical forms of American tegumentary leishmaniasis. Equal amounts of extracellular vesicle (EV) proteins obtained from L. braziliensis isolates associated with cutaneous leishmaniasis (CL), disseminated leishmaniasis (DL), and mucosal leishmaniasis (ML) were resolved on a 10% SDS-polyacrylamide gel. Soluble Leishmania antigen (SLA), prepared from whole-parasite lysates, was included as a reference. Lane M: molecular weight marker. The arrow indicates the protein region around 40 kDa. Representative image from independent EV isolations.

Crucially, this comparative analysis provides direct structural evidence that our EV preparations contain unique molecules that are not as abundant in SLA. This marked biochemical divergence substantiates our immunological premise. By establishing that these EVs carry exclusive molecular targets, we suggest that the antibodies in the serum are reacting specifically to these unique molecules, thereby properly characterizing the presence and activity of the IgGs responsive to parasite-derived EVs.

### 3.7 EV stimulation does not significantly alter B cell subset distribution in CL patients

To assess whether *L. braziliensis* EVs directly modulate B cell phenotypes, peripheral blood mononuclear cells (PBMCs) from CL patients were stimulated with EVs derived from CL, ML, or DL isolates in the presence or absence of soluble *Leishmania* antigen (SLA).

Flow cytometric analysis revealed no significant differences in the frequency of circulating B cell subsets defined by CD19, CD24, and CD38 expression among the different stimulation conditions (**Supplementary Data**). These results indicate that short-term exposure to parasite-derived EVs does not significantly alter B cell subset distribution *in vitro*, suggesting that EV-induced systemic humoral responses are unlikely to be driven by acute modulation of B cell differentiation.

## 4 Discussion

In the present study, we investigated systemic humoral responses against *Leishmania braziliensis*–derived EVs in ATL patients using independent comparative analyses. First, serum IgG reactivity against EVs was significantly higher in patients with CL than in HS, demonstrating that active cutaneous infection is associated with detectable anti-EV systemic antibody responses. In a second analysis comparing clinical forms, patients with CL and DL exhibited higher anti-EV IgG levels than patients with ML, indicating that humoral responses against EV surface antigens differ across the ATL spectrum. Finally, when ECL was evaluated relative to classical CL, anti-EV IgG levels were lower in ECL, consistent with reduced systemic humoral reactivity at early stages of cutaneous disease.

A particularly intriguing finding of this study is the reduced anti-EV IgG levels observed in ML patients compared to CL and DL, despite the well-established association of ML with heightened immune activation and severe immunopathology. Previous studies have consistently demonstrated elevated levels of anti-*Leishmania* IgG and IgE antibodies in ML, including increased anti–SLA IgG levels associated with disease severity (36, 37). These observations appear, at first glance, to contrast with our findings. However, this apparent discrepancy likely reflects fundamental differences in antigen composition and compartmentalization. SLA represents a heterogeneous pool of soluble parasite proteins, whereas EVs constitute membrane-bound structures enriched in a specific subset of parasite-derived molecules, including surface proteins, lipids, and associated cargo (29, 38, 39). Thus, humoral responses against EVs may capture immunological processes distinct from those reflected by SLA-based serology.

The analysis of EV-specific IgG responses in patients with ECL provides insight into the temporal development of humoral immunity during *L. braziliensis* infection. Here in, anti-EV IgG levels were lower in ECL than in classical ulcerated CL, indicating that systemic EV-reactive antibody responses are less pronounced at early stages of disease and increase as cutaneous infection progresses. ECL is clinically defined by recent infection and non-ulcerated lesions and has been associated with early immune activation that is predominantly localized, frequently accompanied by regional lymphadenopathy, with limited systemic immune manifestations (40, 41). In this context, the lower IgG reactive to EVs levels observed in ECL likely reflect reduced systemic antigen exposure at early stages of infection, supporting the notion that humoral responses against *L. braziliensis* EVs accumulate over time in association with lesion progression and sustained host–parasite interaction.

A notable finding of this study is the strong positive correlation between IgG responses directed against EVs derived from *L. braziliensis* isolates associated with distinct clinical forms. Although EVs obtained from parasites isolated from CL, ML, and DL patients may differ in their biological activity (unpublished data), our data indicate that they elicit highly correlated systemic humoral responses. This observation suggests the presence of shared antigenic targets among EVs derived from different isolates. Importantly, the ELISA assays employed in this study were performed using intact EVs. Consequently, antibody recognition is restricted to antigens exposed on the vesicle surface, predominantly reflecting immune recognition of membrane-associated proteins and lipids, rather than components contained within the vesicle lumen.

The association between humoral response against EV and therapeutic outcome underscores the clinical relevance of these findings. Higher levels of anti-CL EV and anti-ML EV IgG measured prior to treatment were associated with clinical cure at day 60, whereas lower antibody levels were observed in patients who failed antimonial therapy. Although the predictive performance of anti-EV IgG was moderate, these data suggest that EV reactive IgG levels may reflect underlying immune states that influence early treatment response. Notably, this association was not maintained at day 90, indicating that these IgG levels are more closely linked to early healing dynamics than to definitive lesion resolution. Consistent with this interpretation, previous studies in CL have shown that baseline immune signatures are associated with therapeutic response or failure, while additional immunological and transcriptional pathways—particularly those related to cytotoxicity and tissue damage—are more strongly associated with longer-term outcome (42, 43).

Despite the robust systemic humoral responses observed *in vivo*, short-term *in vitro* stimulation of PBMCs with parasite-derived EVs did not result in detectable changes in the frequency of circulating B cell subsets (**Supplementary Data**). This negative finding should be interpreted with caution, as the experimental design was not intended to model prolonged antigen exposure or lymphoid microenvironments required for B cell activation and differentiation. Rather than indicating a lack of B cell involvement, these results suggest that antibody responses against EVs observed in patients are unlikely to arise from acute, direct modulation of circulating B cells, and may instead reflect integrated immune processes occurring *in vivo* overtime, including sustained antigen exposure and interactions within secondary lymphoid tissues.

Taken together, our results identify parasite-derived EVs as immunologically relevant structures during *L. braziliensis* infection and reveal distinct patterns of humoral immunity reactive to EVs across clinical forms of ATL. The dissociation between anti-EV and anti-SLA IgG responses in ML underscores the importance of antigen compartmentalization in shaping humoral immunity. Furthermore, the early induction of anti-EV antibodies in ECL and their association with therapeutic outcome in CL suggest that EV-based serological markers may provide complementary information to existing diagnostic and prognostic tools.

## Data Availability

The raw data supporting the conclusions of this article will be made available by the authors, without undue reservation.

## Supplementary material

Supplementary Figure 1 Correlation of serum (A) αCL EV, (B) αML EV, and (C) αDL EV IgG levels with healing time; and (D) αCL EV, (E) αML EV and (F) αDL EV IgG levels with lesion size. Statistical analysis was performed with Pearson correlation.

Supplementary Figure 2 Stimulation with *Leishmania braziliensis*–derived extracellular vesicles does not alter circulating B cell subset distribution. Peripheral blood mononuclear cells (PBMCs) from patients with cutaneous leishmaniasis (CL) were stimulated *in vitro* with extracellular vesicles (EVs) derived from *Leishmania braziliensis* isolates associated with cutaneous leishmaniasis (CL), mucosal leishmaniasis (ML), or disseminated leishmaniasis (DL), in the presence or absence of soluble *Leishmania* antigen (SLA). After 72 h of culture, B cell populations were analyzed by flow cytometry. (a) Representative gating strategy used to identify total B cells (CD19^+^) and B cell subsets based on CD24 and CD38 expression. (b) Frequency of circulating B cell subsets under each stimulation condition. Data are shown as individual values with median and interquartile range. Statistical analyses were performed using the Wilcoxon matched-pairs signed-rank test. No statistically significant differences were observed among stimulation conditions.
